# An effective evolutionary algorithm for protein folding on 3D FCC HP model by lattice rotation and generalized move sets

**DOI:** 10.1186/1477-5956-11-S1-S19

**Published:** 2013-11-07

**Authors:** Jyh-Jong Tsay, Shih-Chieh Su

**Affiliations:** 1 Department of Computer Science and Information Engineering, National Chung Cheng University, 168 University Road, Minhsiung Township, Chiayi County 62102, Taiwan

## Abstract

**Background:**

Proteins are essential biological molecules which play vital roles in nearly all biological processes. It is the tertiary structure of a protein that determines its functions. Therefore the prediction of a protein's tertiary structure based on its primary amino acid sequence has long been the most important and challenging subject in biochemistry, molecular biology and biophysics. In the past, the HP lattice model was one of the ***ab initio ***methods that many researchers used to forecast the protein structure. Although these kinds of simplified methods could not achieve high resolution, they provided a macrocosm-optimized protein structure. The model has been employed to investigate general principles of protein folding, and plays an important role in the prediction of protein structures.

**Methods:**

In this paper, we present an improved evolutionary algorithm for the protein folding problem. We study the problem on the 3D FCC lattice HP model which has been widely used in previous research. Our focus is to develop evolutionary algorithms (EA) which are robust, easy to implement and can handle various energy functions. We propose to combine three different local search methods, including lattice rotation for crossover, K-site move for mutation, and generalized pull move; these form our key components to improve previous EA-based approaches.

**Results:**

We have carried out experiments over several data sets which were used in previous research. The results of the experiments show that our approach is able to find optimal conformations which were not found by previous EA-based approaches.

**Conclusions:**

We have investigated the geometric properties of the 3D FCC lattice and developed several local search techniques to improve traditional EA-based approaches to the protein folding problem. It is known that EA-based approaches are robust and can handle arbitrary energy functions. Our results further show that by extensive development of local searches, EA can also be very effective for finding optimal conformations on the 3D FCC HP model. Furthermore, the local searches developed in this paper can be integrated with other approaches such as the Monte Carlo and Tabu searches to improve their performance.

## Background

Proteins are essential biological molecules which play vital roles in nearly all biological processes. It is the tertiary structure of a protein that determines its functions [1-3]. Therefore the prediction of a protein's tertiary structure based on its primary amino acid sequence has long been the most important and challenging subject in biochemistry, molecular biology and biophysics. Although the interaction between individual atoms can be calculated to model the folding of a protein in a search of the tertiary structure at the lowest free energy, the massive degree of computational complexity makes this approach infeasible. As a result, researchers have proposed to develop simplified models to reduce the computational complexity in modelling protein 3D structure.

Lau and Dill [4] proposed a simple Hydrophobic-Polar model (HP model) based on the hydrophobic interaction between amino acids which has greatly reduced the complexity involved in protein structure prediction. The HP model has thus been used by many researchers and has been applied in various lattice algorithms [5] such as 2D Square [4,6-14], 2D Triangular [15-18], 3D Cubic [13,19,20], 3D Triangular [21] and 3D *Face-Centered Cubic *(FCC) [22-28] lattices. Although algorithms on simplified HP lattice methods did not achieve high resolution, they provided a macrocosm-optimized protein structure. The model has been employed to investigate general principles of protein folding as well as to predict protein tertiary and quaternary structure.

Although the HP lattice model has greatly reduced the complexity of the protein folding problem, it is still NP-hard [29-31]. The evolutionary algorithm is one of the major methods used to investigate protein folding. It is so far the most widely used approach in protein folding simulation [32]. Unger and Moult [14] presented a pioneering work which proposed the first Genetic Algorithm (GA) developed from Evolutionary Programming to solve protein folding problem in the 2D HP model. Their work has had a wide impact in the early progress of computational protein folding. Later, Jiang *et al.*, [11] combined GA with the Tabu search and demonstrated that combinatorial genetic algorithms performs better than a single GA. Recently, Hoque *et al.*, [7,22] proposed a twin-removal strategy to maintain the diversity of chromosomes to improve the performance of GA.

Various local search methods have been proposed to improve the search performance of evolutionary algorithms. Most of them are based on the concept of Move Set [7,10,11,15,20,22,33]. Dill *et al*. [34] proposed Three-Bead and End Flip for single-point move and Crankshaft for double-point move. Lesh *et al.*, [12] developed Pull Move, and showed that Pull Move is a very effective local search method. Thachuk *et al.*, [20] proposed End Move, Corner Move [35] and Crankshaft Move [36] to compensate for the disadvantages of Pull Move. It was shown that their approach performed better than the most advanced Ant Colony Optimisation (ACO) [13] and pruned-enriched Rosenbluth method (PERM) [37] on 2D square and 3D cubic models. Hoque *et al.*, [7,22] also used similar local search strategies in GA such as Pull Move, Diagonal Move and Tile Move. Sali *et al*. [38] and Mann *et al*., [39] proposed a K-local move that can give sufficient structural changes within a successive interval of fixed length K. Huang *et al*. [10] proposed a Genetic algorithm based on optimal secondary structures (GAOSS) in which the authors designed three types of 2D structural motifs in the 2D square lattice model to improve the efficiency and increase the search capacity. The approach of Huang *et al*., involves a move set method based on special motifs.

Rotation is another transformation which has been proposed by Unger and Moult [14] to increase the successful rate of crossovers and mutations. However, rotation has been mainly applied to structures such as square and Triangular [17] lattices, but less explored in other lattice structures, including FCC lattices. This may be partly because how to perform rotation in them is not as clear as in cubic lattices.

In this paper, we propose to study the effect of lattice rotation in search of optimal conformations on lattice models. We focus on 3D FCC lattice which gives higher degree of freedom and does not involve the parity problem appearing in cubic lattice [21]. This model has the highest packing density [40] and can render conformations closer to the real or high resolution folding [41]. We aim to develop effective EA-based approaches which combine lattice rotations and move set operations. We have proposed three different local search methods, including lattice rotation for crossover, K-site move for mutation, and generalized Pull Move. These three methods form our key components to improve EA-based approaches. Experiment shows that our approach performs better than previous EA-based approaches. In addition, our approach does not rely on any specific form of mathematical optimization so that it is robust and can handle arbitrary energy functions and be integrated with other approaches such as Monte Carlo and Tabu search to improve their performance.

It should be noted that, to this date, constraint programming (CP) [23,24,27] is the state-of-the-art method which performs best for protein folding on HP lattice model [28]. This approach can ensure the solution to be the global optimum. However, from our experience with HPstruct [23,24] which is an excellent tool based on constraint programming, CP-based approaches do not always converge to return optimal conformations. In addition, it is difficult, if not impossible, to modify CP-based approaches to handle complex energy functions efficiently, such as energy functions of pairwise interactions among all 20 amino acids [33,42,43]. On the contrary, EA-based approaches are robust and not constrained by any specific form of energy function. Although experiment shows that CP-based approaches such as HPstruct achieve the best performance [23,24], provided they converge, we still need complementary methods such as EA-based approaches to compensate for their disadvantages, especially when they fail to converge.

The remainder of this paper is organized as follows. Section 2 describes preliminaries, and reviews the HP model and 3D FCC lattice. Section 3 presents the proposed approaches, and gives details for main components of our algorithm, including rotation-based crossover, K-site-move-based mutation and generalized Pull Move. Section 4 explains the experimental results. Section 5 concludes and discusses future work.

## Preliminaries

In this section, we review the HP model, 3D FCC lattice and fitness function which are used in our approach.

### HP model

**Figure 1:**
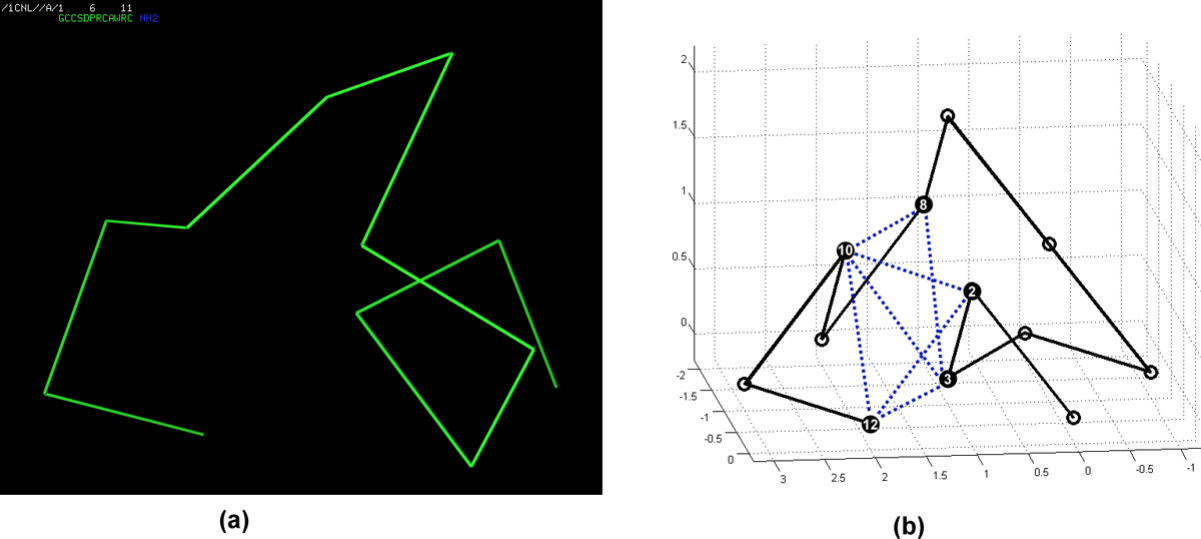
**A ground-state conformation in the 3D FCC HP model**. An example on HP lattice model: (a) The native state of the protein with PDB id 1CNL, (b) an optimal HP conformation for 1CNL on 3D FCC lattice with 7 HH contacts denoted by dashed blue lines.

The HP lattice model is the most frequently used simplified model and is based on the observation that the hydrophobic interaction between the amino acid residues is the driving force for the protein folding and for the development of native state in proteins [4]. In this model, a protein is represented as a linear chain of *n *amino acids. Each amino acid is classified based on its hydrophobic characteristics as an H (hydrophobic or non-polar) or a P (hydrophilic or polar). The HP lattice model allows a chain conformation to be represented as a self-avoiding walk (SAW) on the lattice path favouring an energy-free state due to HH interaction. HH interaction in this study refers to 'topological neighbours' and not to the 'connected neighbours' as in the above mentioned chain. Figure 1 gives a conformation on a 3D FCC lattice for protein 1CNL with HP-sequence "PHHPPPPHPHPH". The number of HH contacts in Figure 1(b) is 7.

### 3D FCC lattice

**Figure 2:**
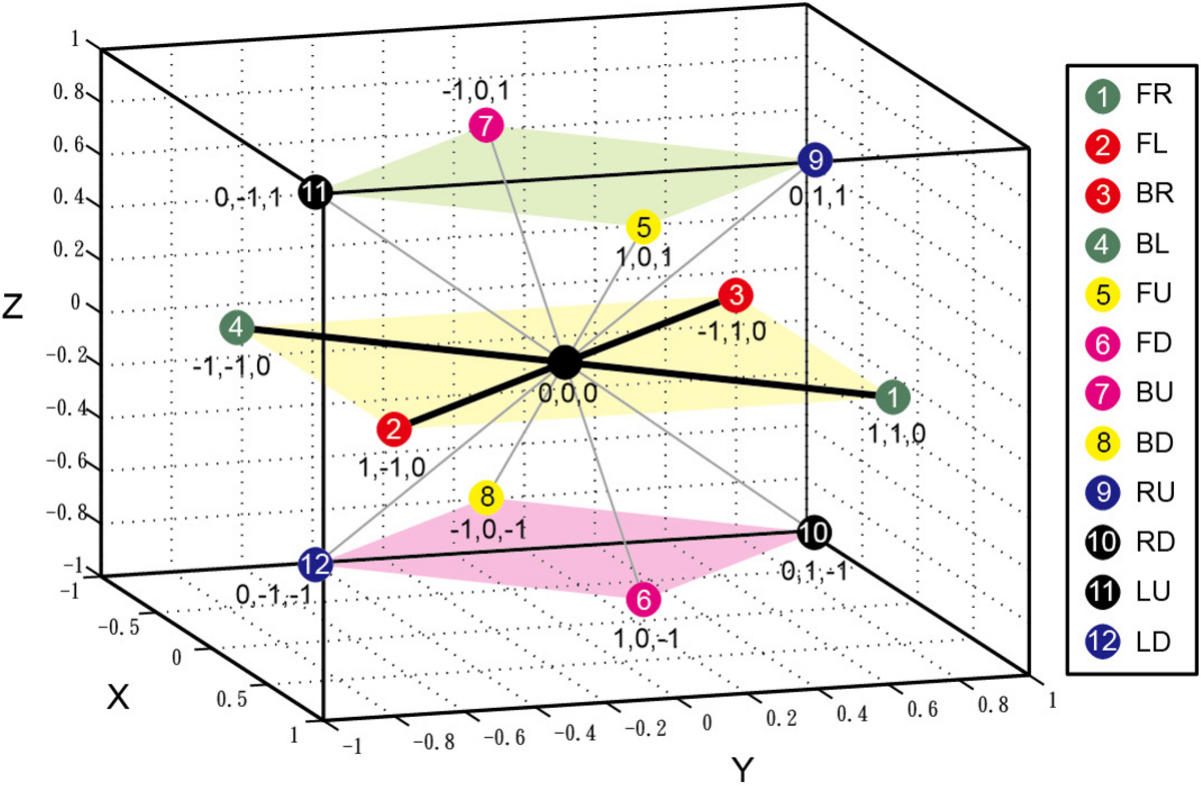
**The FCC lattice model: each lattice point has 12 neighbours**.

Raghunathan and Jernigan made an effort in 1997 to find and define a basic unit for the 3D arrangement surrounding one amino acid [40]. Consequently, a 3D FCC model was proposed and developed as shown in Figure 2. This model can produce a nearly perfect angular distribution for the amino acids and therefore can be used directly to generate amino acid chains. In this model there are 8 cubes with 14 faces and 12 vertices, which is a unique convex polyhedral containing regular polygons, triangles and squares. As a result, every lattice point will have 12 neighbours.

In a FCC lattice, we can define the domain as the set of points(*x, y, z*) ∈ *Z *so that *x *+ *y *+ *z *is even. Two FCC points *p_i _*= (*x_i_, y_i_, z_i_*) and *p_j _*= (*x_j_, y_j_, z_j_*) are *adjacent *if and only if |*x_i _*− *x_j_*| ≤ 1, |*y_i _*− *y_j_*| ≤ 1, |*z_i _*− *z_j_*| ≤ 1 and |*x_i _*− *x_j_*| + |*y_i _*− *y_j_*| + |*z_i _*− *z_j_*| = 2 [33]. Each FCC lattice point is adjacent to 12 neighbouring points, and three consecutive adjacent points form one of these four angles 60°, 90°, 120° and 180°. In this paper, the 12 neighbours of each lattice point are labelled as numbers from 1 to 12, where 1 is for *FL*(+1,+1,0), 2 for *FR*(+1,-1,0), 3 for *FU*(-1,+1,0), 4 for *FD*(-1,-1,0), 5 for *BL*(+1,0,+1), 6 for *BR*(+1,0,+1), 7 for *BU*(-1,0,+1), 8 for *BD*(-1,+0,-1), 9 for *LU*(+0,+1,+1), 10 for *LD*(+0,+1,-1), 11 for *RU*(+0,-1,+1), and 12 for *RD*(+0,-1,-1). Symbols *FL, FR, FU, FD, BL, BR, BU, BD, LU, LD, RU *and *RD *are used to denote fold directions with *FL *for front-left, *FR *for front-right, *FU *for front-up, *FD *for front-down, *BL *for back-left, *BR *for back-right, *BU *for back-up, *BD *for back-down, *LU *for left-up, *LD *for left-down, *RU *for right-up and *RD *for right-down. The vector following each symbol is its corresponding direction vector.

A conformation is a sequence of adjacent points in the lattice and can be encoded as a sequence of numbers from 1 to 12. Two hydrophobic amino acids *x_i _*and *x_j _*and in lattice positions *p_i _*and *p_j _*respectively are said to be in *HH contact*, that is contact(*p_i_, p_j_*) = 1, if and only if they are adjacent in the lattice, but not if they are adjacent in the primary sequence where *x_i _*− *x_j_*| + |*y_i _*− *y_j_*| + |*z_i _*− *z_j_*| = 2 and |*i *- *j*| > 1. A conformation is valid if it consists of a self-avoiding walk (SAW) in the lattice: that is where *p_i _*≠ *p_j _*for *i *≠ *j*. Otherwise, it is invalid.

### Fitness function

Assume each HH contact contributes energy -1 to the conformation. The free energy of a protein conformation is defined as the negative sum of its HH contacts as follows. Let *s*=*s*_1_*s*_2 _... *s_n _*be an HP sequence, and c=*p*_1_*p*_2_...*p_n _*be a valid conformation for *s*. Then the free energy *E(c) *of *c *is defined as follows:

(1) $$E\left(c\right)=\sum_{i=1}^{n-2}\sum_{j=i+2}^{n}contact\left(p_{i},p_{j}\right)$$

Hence, the problem of protein folding is formulated as an optimization problem which aims to find the conformation with minimal free energy. That is to find c^* ^∈ *C *(s) such that *E*(c^*^) = min*{E(c)*|*c *∈ *C}*, where *C*(*s*) is the set of all valid conformations for *s *[13].

## The proposed method

**Figure 3:**
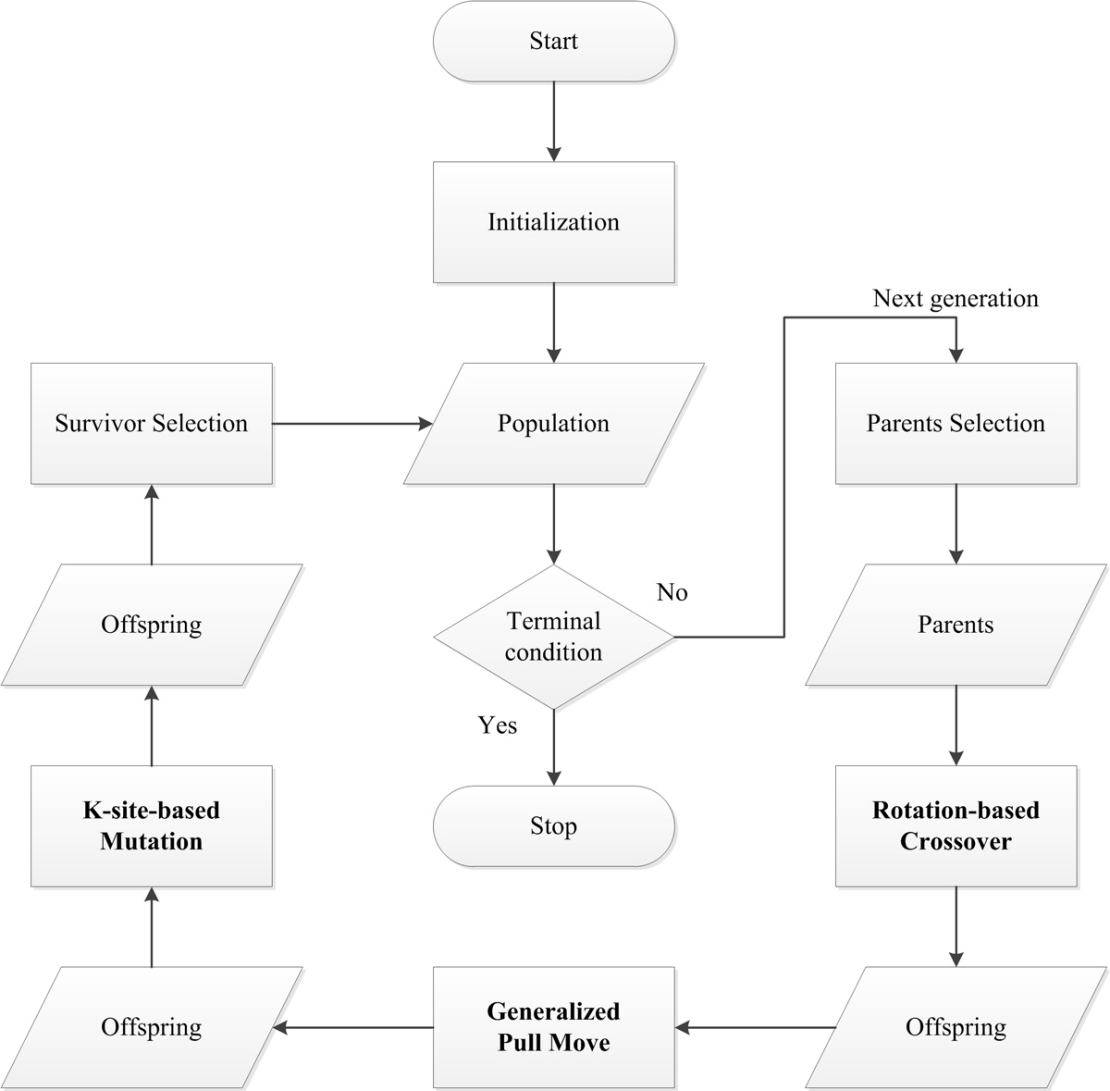
**Main steps of the proposed EA-based approach**.

In this section we present the proposed EA-based approach. Figure 3 shows the main step. To improve the search performance, the proposed approach enhances crossover by lattice rotation, Pull Move by generalized Pull Move and mutation by K-site move. We next explain details of each main step.

### Initialization

An initial population was generated randomly from an *n *- 1 dimensional space within a fixed range. We apply Depth-first search [6,8] to generate random conformations. Each chromosome in the population needs to be evaluated for its fitness value as defined in equation (1). Our objective is to minimize the fitness value; that is, to maximize the number of HH contacts. The evaluated chromosomes were sorted according to their fitness values. This sorted population served as the basis of subsequent reproduction processes.

### Parent selection

Parent selection is the process of collecting chromosomes to be selected as parents for crossover. We apply the tournament selection method in which the better of two randomly selected chromosomes is selected as one parent.

### Rotation-based crossover

Crossover is a process of taking two parent conformations and producing child conformations from them. Several different crossover methods have been proposed. We use the simplest 1-point crossover in which a single crossover point on both parents' conformations is selected, and all data beyond that point in either conformation are swapped between the two parent conformations. The resulting two conformations are the children. However, crossover may fail to produce legal child conformations as child conformations may violate the SAW constraint, i.e. points in a child conformation may overlap. In order to increase the successful rate of crossover, we develop a rotation-based crossover in which parts from parent conformations are rotated at various angles to produce child conformations. Notice that rotation-based crossover was first proposed by Unger and Moult [14] on 2D square lattice. In this paper, we apply it to 3D FCC lattice model. We investigate the geometric structure of 3D FCC lattice, and identify several valid rotations which keep all rotated points fully overlapped with the original points in the lattice, and can be performed by simple neighbour permutations. In particular, we identified 17 rotations which are classified into two types, square-based and triangle-hexagon-based. Thus, each rotation-based crossover will generate at most 17 new chromosomes. Each rotation is performed by first partitioning all lattice points into parallel planes, and then rotating all planes synchronously.

**Figure 4:**
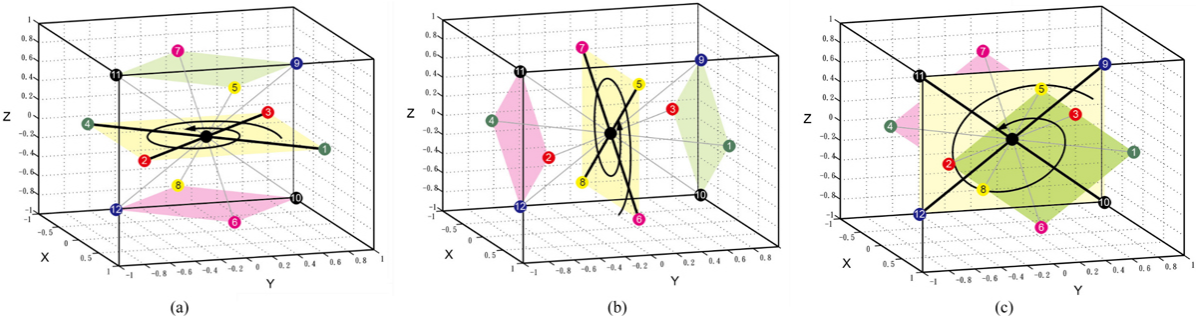
**Square-Based Rotation: 3 ways to partition the 12 neighbours of the central point into 3 squares**.

In square-based rotation, the neighbours of the central point are partitioned into squares. Figure 4 shows 3 different partitionings of the 12 neighbours into 3 squares. For each partitioning, we rotate each square synchronously. The rotation axis is the line defined by the centres of the 3 squares, and the rotation angle is one of 90°, 180° or 270°. We thus can define 9 different square-based rotations.

**Figure 5:**
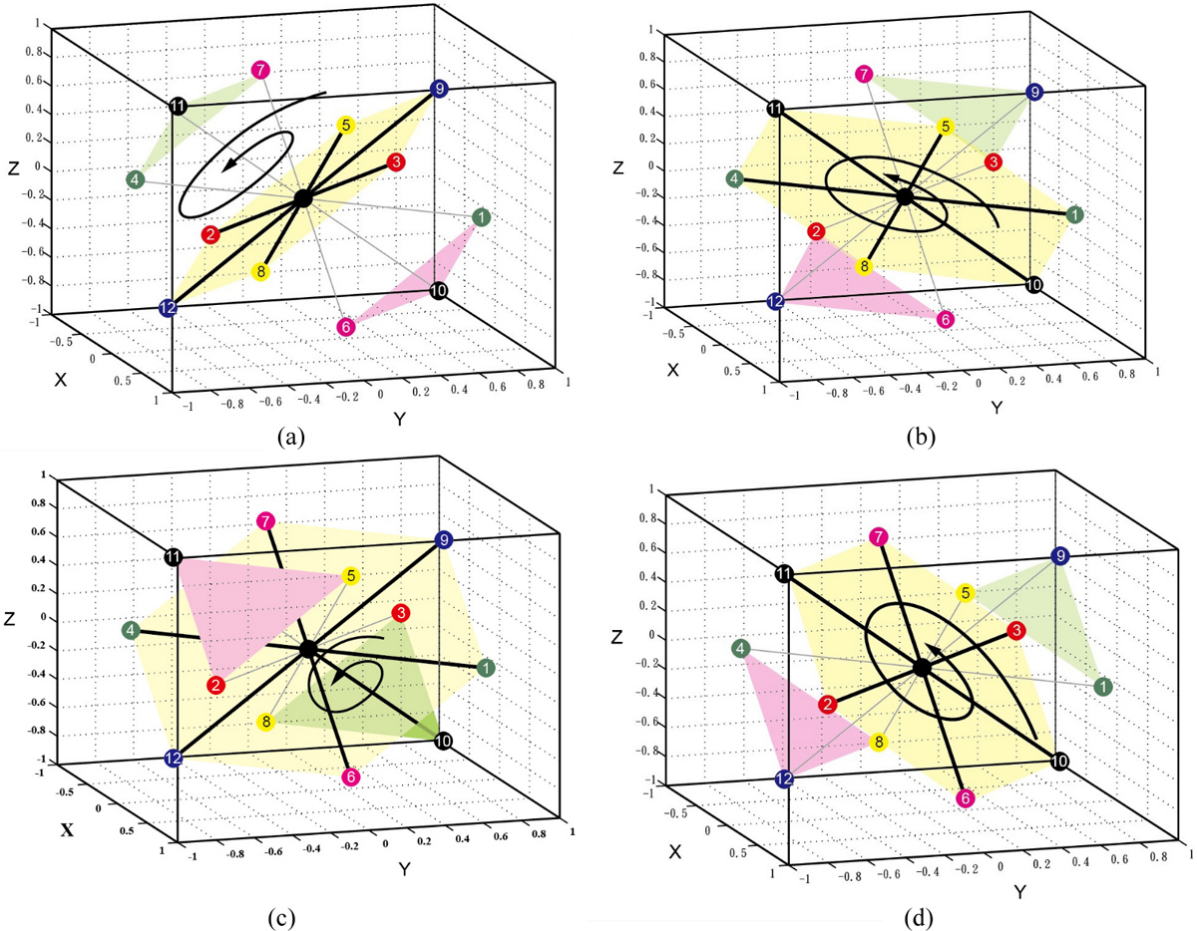
**Triangle-Hexagon-Based Rotations**. Triangle-Hexagon-Based Rotations: 4 ways to partition the 12 neighbours of the central point into two triangles and one hexagon.

In triangle-hexagon-based rotation, the 12 neighbours of a lattice point are partitioned into two triangles and one hexagon. Figure 5 shows 4 different partitionings for triangle-hexagon-based rotation. The rotation axis is the line defined by the centres of the two triangles and the hexagon, and the rotation angle is 120° or 240°. Thus 8 different triangle-hexagon-based rotations are defined.

**Figure 6:**
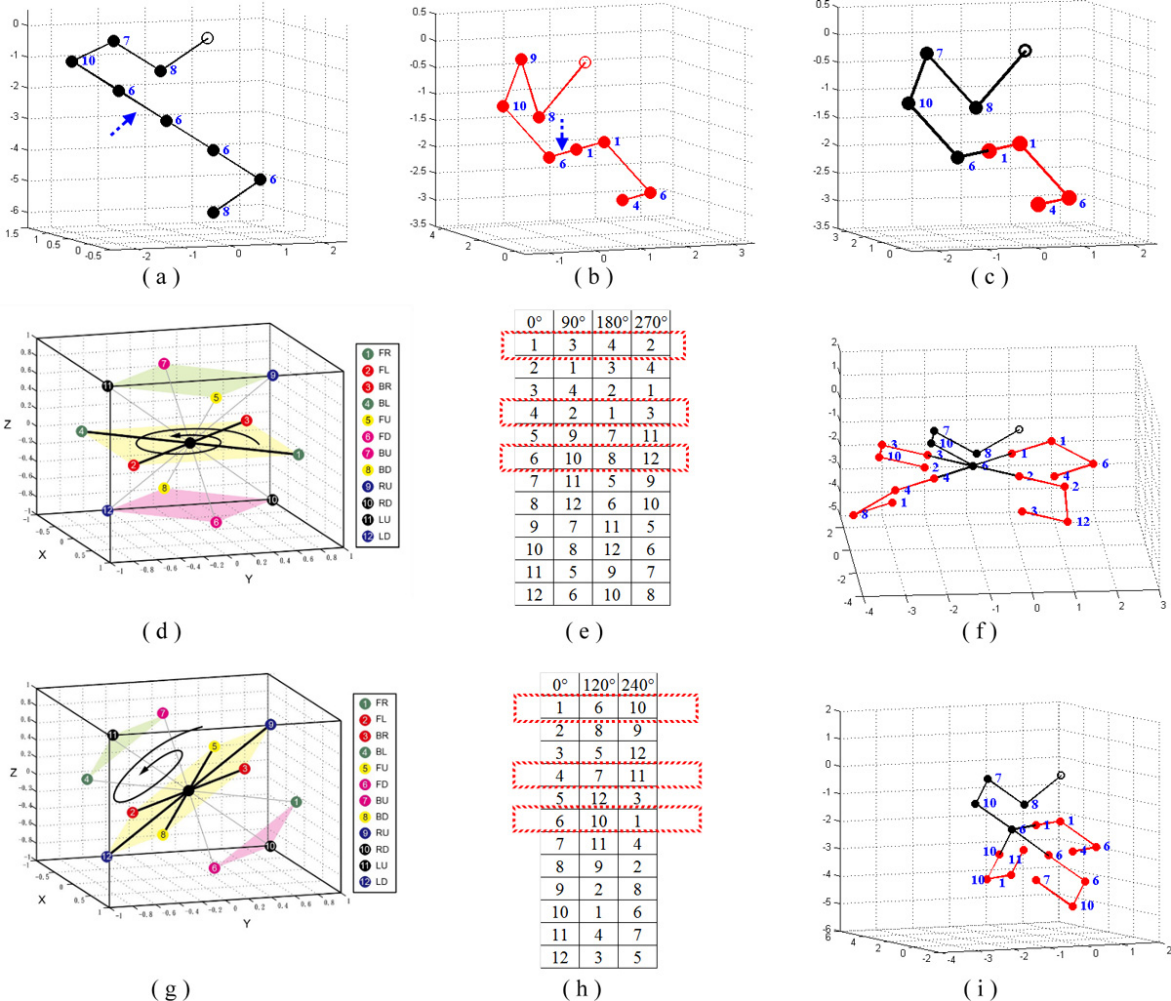
**Rotation-based crossover operate**. Illustration of rotation-based crossover: (a) and (b) are the parent conformations; (c) is the offspring without rotation; (d) shows the 3 squares in one partitioning of neighbours; (e) shows the corresponding label permutation for rotation angle 90°, 180° and 270°; (f) shows the 4 offspring with the part in red rotated 0°, 90°, 180° and 270°; (g), (i) and (j) illustrate triangle-hexagon-based rotation.

Note that, for each above-mentioned rotation, the representation of a rotated conformation can be computed from its original conformation by label permutation. Figure 6 gives an illustration of square-based rotations and their corresponding label permutations. In Figure 6(a) and 6(b) give the parent conformations. Figure 6(c) gives the offspring without rotation. Figure 6(d) shows the 3 squares in the partitioning of neighbours. Figure 6(e) gives the corresponding label permutations for rotation angles90°, 180°and 270° respectively. Figure 6{f) gives the 4 offspring with the part in red rotated 0°, 90°, 180°and 270° respectively. Note that the label sequence of the conformation of the red part for each rotation angle is (1,1,6,4) for 0°, (3,3,10,2) for 90°, (4,4,8,1) for 180° and (2,2,12,3) for 270°. Each rotated conformation is computed by its corresponding label permutation in Figure 6(e). An illustration of triangle-hexagon-based rotation is given in Figure 6(g), 6(i) and 6(j).

### Generalized pull move

Pull Move was first proposed by Lesh *et al.*, [12] and used as local search on the 2D square HP protein folding problem. Böckenhauer *et al.*, [15] further applied Pull Move in 2D triangular and 3D FCC lattice models and demonstrated that this method is reciprocal and complete.

**Figure 7:**
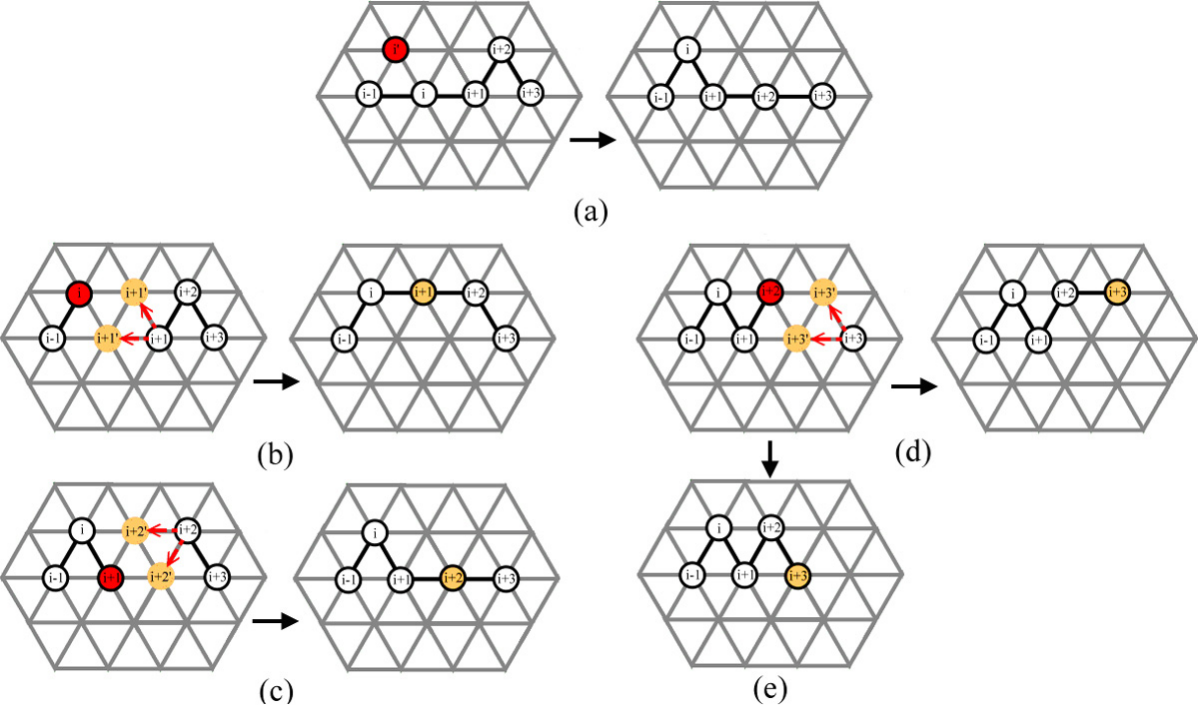
**Generalized Pull Move**. Generalized Pull Move on 3D FCC lattice: (a) shows the result obtained by the traditional Pull Move; (b) to (e) shows the 4 possible results obtained by Generalized Pull Move.

In Pull Move, the next point is pulled to the original position of its previous point. In this paper, we propose a Generalized Pull Move (GPM) in which a point is not restricted to being moved to the position of its previous point; instead it can be moved to any common neighbour of the new position of its previous point and its current position. We thus can have multiple choices to move the next point. Figure 7 gives an illustration of GPM on 2D FCC. Figure 7(a) shows the only result obtained by Pull Move, and Figure 7(b)-(e) demonstrates the 4 possible results obtained by GPM. It is noted that in GPM, after the *i*th point is moved, there are 2 possible positions to move the *(i+1)*th point to as there are 2 common neighbours between the new position of the *i*th point and the original position of the *(i+1)*th point. On 3D FCC, the number of possible positions to move to the next point is 4.

When GPM initiates, if the number of possible choices to move the next point to is greater than one, then a random choice is made. If the free energy of the newly pull-moved conformation is lower than the original conformation, the new conformation will replace the original conformation. Otherwise, the original conformation will remain unchanged.

### K-site-move-based mutation

Monomer or dimer moves were often used in past research as methods for local search. In this paper, we apply K-site move to enhance mutation to search the best conformation obtained by moving *K *consecutive points in the conformation.

However, the searching space increases exponentially with the increase of K because the number of possible SAWs in a 3D FCC lattice is given by *SAW_FCC _*= 1.26*K*^0.16 ^(10.0364)*^K ^*[44]. In the implementation, a lower bounding technique is applied to reduce the search space. In particular, for each search path, a lower bound, which is defined as the sum of the path length and the Euclidean distance between its end point of the path and the destination point, is estimated and the path is pruned in the search process if the estimated lower bound is larger than K+1. Note that a small value of K may limit the search space and degrade the effectiveness of the search process. On the other hand, a large value of K can enlarge the search space but, at the same time, increase the search time exponentially. The value of K is set as 3 in this paper.

### Survivor selection

After generating a set of offspring, only the top fittest chromosomes are selected to survive into the next generation.

### Termination

The process is repeated a fixed iteration size of times. When terminated, the best conformation remaining in the population is returned.

## Experimental results

To evaluate the effectiveness of our approach, experiments over 4 data sets were carried out, including two sets of short amino acids HP-sequences with lengths from 20 to 64, and two sets of longer amino acids HP-sequences with lengths from 90 to 200. Tables 1, 2, 3 and 4 summarize amino acids HP-sequences of the 4 data sets.

**Table 1:** Data Set I: a group of eight HP sequences with 20-64 amino acids.

**Seq**.	**Len**.	Protein Sequence
S1	20	HPHPPHHPHPPHPHHPPHPH
		
S2	24	HHPPHPPHPPHPPHPPHPPHPPHH
		
S3	25	PPHPPHHPPPPHHPPPPHHPPPPHH
		
S4	36	PPPHHPPHHPPPPPHHHHHHHPPHHPPPPHHPPHPP
		
S5	48	PPHPHHHPHHHPPPPPHHHHHHHHHHPPPPPPHHPPHHPPHPPHHHHH
		
S6	50	HHPHPHPHPHHHHPHPPPHPPPHPPPPHPPPHPPPHPHHHHPHPHPHPHH
		
S7	60	PPHHHPHHHHHHHHPPPHHHHHHHHHHPHPPPHHHHHHHHHHHHPPPPHHHHHHPHHPHH
		
S8	64	HHHHHHHHHHHHPHPHPPHHPPHHPPHPPHHPPHHPPHPPHHPPHHPPHPHPHHHHHHHHHHHH

**Table 2:** Data Set II

**Seq**.	**Len**.	Protein Sequence
H1		HPHHPPHHHHPHHHPPHHPPHPHHHPHPHHPPHHPPPHPPPPPPPPHH
H2		HHHHPHHPHHHHHPPHPPHHPPHPPPPPPHPPHPPPHPPHHPPHHHPH
H3		PHPHHPHHHHHHPPHPHPPHPHHPHPHPPPHPPHHPPHHPPHPHPPHP
H4		PHPHHPPHPHHHPPHHPHHPPPHHHHHPPHPHHPHPHPPPPHPPHPHP
H5	48	PPHPPPHPHHHHPPHHHHPHHPHHHPPHPHPHPPHPPPPPPHHPHHPH
H6		HHHPPPHHPHPHHPHHPHHPHPPPPPPPHPHPPHPPPHPPHHHHHHPH
H7		PHPPPPHPHHHPHPHHHHPHHPHHPPPHPHPPPHHHPPHHPPHHPPPH
H8		PHHPHHHPHHHHPPHHHPPPPPPHPHHPPHHPHPPPHHPHPHPHHPPP
H9		PHPHPPPPHPHPHPPHPHHHHHHPPHHHPHPPHPHHPPHPHHHPPPPH
H10		PHHPPPPPPHHPPPHHHPHPPHPHHPPHPPHPPHHPPHHHHHHHPPHH

**Table 3:** Data Set III

**Seq**.	**Len**.	Sequences
F90_1	90	PPHHHPPPHHPPPPHHPHHHHHHPHPHPHHPHHHHHPHHHPHPHHHHP HHPPPPHHHPHPHPPHHHPHHPHPHPPHHHPPPPHHPPHPPP
		
F90_2	90	PHHPPHPHHPHHHPHHHPPHHHHHHPPHPHPPPPHHHPHPPHHHHPHH HHPHHHPHHPPPPPHHPPPPHPHPHPHPHHPPHHHPPPHHHP
		
F90_3	90	HPHPHHHPHHHHPHHHPPPHPPPHPPPPHHHPPHPPPPHHHPPPPPPPPHP HHPHHHHPHHHPHPHHPPHHHHHPHHPPHHPHHHHHHPH
		
F90_4	90	PHHHPPHPPHPHPPPPHPPPHPHPPHPHHPHPPPHHHPHHHPPHHHPPHPP PPHPHHHPPHHPPHHHPPHHHHHHPHHHHHHHPHHHHPH
		
F90_5	90	PPPHPHHHHHHHPPPHPPHHHHHPHHPPHHPPHHHHPHPHPHHPPHHPPP PHPPPHHHPHPHHHHHHHPHHPHPPHHPPPHHHPHPPHPP

S1	135	HHHHPHHHHHHPPHHPHHHHHHHHPHHPHHHHHHHHHHPPHHPPPPPH HPHHHHHHHHPHPPHHPPPHHHHHHHHPHHHHHHPPHHHHHHHPHPPH HHHHHHHHPPHHPPPHHHHHHHPHHPHHHHHHHPPHHHH
		
S2	151	HHPPHPHHHHHHHHHHPHPPPPHHHPPPHHHHHPPHHHHHPPHHHHPPH HHHPPHHHHHHPHHHHPPPHHPPPHHHHHHHHPHPPHHHPPPHHHHHPP HHHHHHHPPPHHPPHHHHHPPPHHHHHHHHHPHPPHHHHHHHPPPHHH PPHHP
		
S3	162	HHHPPPHHPHHPPPPPHHHHHHHHPHPPHHPHHPHHHHHPPPHHHHHHH HHPPHPHPPHPHPPHHHPHPPHPHPPPHHHHHHPHHHHPPPHHHPPPPHH PPPHHHPPHHHHPHHHHHPPHHHHHHPPPHHHHHHPPPHPPHHHHPHHH HHHHPPHHPPHHH
		
S4	164	HHPPHPHHHHHHHPPHPHPPHPHPPPPHHHPPPHHPHPHHPPHHHHHPPH HHHPPHHHHPPHHHHHHPHHHHPPPHHPPPHHHHHHHHPHPPHHHPPPH HHHHPPHHHHHHPHPPPHHPPHHHPHHPPPHPHHHHHHHPHPPHPPHHH HHHPHPPPHHHPPHHP

R1	200	PPPHPHHPHHPPPHPHPPPPHPHHPPHPHHHHHPPHHPPHHHHHHPPHPPH HPPHPHPHHHHHPHHPHHHPPPHHHPHHPPHPHPPHPPPHPPHPPHPPHH HPHHHPHPPHPHHPHHHHPHPHHHPHHHPPPPPPHHHHHHPPPPPPPPHH HPPHPHPPPHPHPHPHHPPHHPPPPHHHHHHPPPHHPPPPPHPPPHHPP
		
R2	200	HPHHPPHPPPPPHHPHPHPHHPPHPPPPHHHHHHPPPHPPHHHPPHPPPPHH PPHHHPHPHHHPPHPHHPPHPHHPPPPHHPPHPPHHHHPPPPPHHHPPPPHP PPPPPHPPHHPHHHHPHHHHHHHHPPHHPPPHPHHHPHHHHHPHHPHHHP HPHHPPPPHPHHPHHHPHPPPPHPPPPPPHPHHHHHPHHPPPHPPH
		
R3	200	HPHHHPHHPHPHPPPHHHHHPHPHPHHHHPPPHHPPPPPPHHPPPPHPHHH PPPPHPPPHHPHHPPPHPPHPPPHHHHPHHPHPPPPHHPPPHHPPHPPPHPPH HHPHHHPHPPHPHHHHPPHHPPPPHHHPHHPPHPPHHHHPPHPHPPHPHPP PPPHPHPHHHHHHHPHPHHHHHHPHHPPPPHPPPPHPPPHHHPHH

F180_1	180	HHPPHHHHHPHHHPPPHHHPPHHHPHPPHHHHHPPPHHHPPPHPHHPPPP PHHPPHHPHHPHPHHPPPPPHHHPPPPHPHHHPPHPPPHHHPHHHHPPHH PHPHHHHPHHHHPPHHPHHPHHPHHHPHPPHPHHPHPHHPHHHPHHPPH PPPHPPPPPPPHHHPHHHHHPHHHHHPPHPP
		
F180_2	180	PHHPHPPPHPPHHPHHHPHPHHPHHHPHHHPPPHHPPHPHPHHPHHHHP PHHPHPHHHHHPHHPPPPHPHPHPPHHHHPHHHHPHHHHHPPHPHHHP PPHPHPPHHPPPHHPHPHPPPPPHPHHPHHHPHPPPPHHPHHHHHPPPHH HHHHHHPHHPPPPPHPPPHPPHPPPHHPHHHHH
		
F180_3	180	HHHPHPPHHPPPHPPPHPHPHPPHHHHPPHHHHHHPHPHHPPPPPHPPHH PHHHHHHHHHHHPPHPPHPPHHHHHHHHPPPPHPPHHHHHPPHHHPPH HPPHHHHHPPPHHHHHHPHHHPPPHHPPHPPPHPPHPPPHPPPPHHHPPH HPHPPHHHPHHPPHHPHHPHPHPHPHPHPHHP

**Table 4:** Data Set IV: the amino acid sequences and the corresponding HP sequences.

**PDB ID**.	**Len**.	Sequences (original and HP transform)
4BP2	123	ALWQFNGMIKCKIPSSEPLLDFNNYGCYCGLGGSGTPVDDLDRCCQTH DNCYKQAKKLDSCKVLVDNPYTNNYSYSCSNNEITCSSENNACEAFIC NCDRNAAICFSKVPYNKEHKNLDKKNC
		
		PHHPHPPHHPHPHPPPPPHHPHPPHPHHHPHPPPPPPHPPHPPHHPPPPPH HPPPPPHPPHPHHHPPPHPPPHPHPHPPPPHPHPPPPPPHPPHHHPHPPPP PHHHPPHPHPPPPPPHPPPPH

2AAS	124	KETAAAKFERQHMDSSTSAASSSNYCNQMMKSRNLTKDRCKPVNTFVH ESLADVQAVCSQKNVACKNGQTNCYQSYSTMSITDCRETGSSKYPNCAY KTTQANKHIIVACEGNPYVPVHFDASV
		
		PPPPPPPHPPPPHPPPPPPPPPPPHHPPHHPPPPHPPPPHPPHPPHHPPPHPP HPPHHPPPPHPHPPPPPPHHPPHPPHPHPPHPPPPPPPHPPHPHPPPPPPPP HHHPHPPPPHHPHPHPPPH

5LYZ	129	KVFGRCELAAAMKRHGLDNYRGYSLGNWVCAAKFESNFNTQATNRNT DGSTDYGILQINSRWWCNDGRTPGSRNLCNIPCSALLSSDITASVNCAKK IVSDGNGMNAWVAWRNRCKGTDVQAWIRGCRL
		
		PHHPPHPHPPPHPPPPHPPHPPHPHPPHHHPPPHPPPHPPPPPPPPPPPPPPH PHHPHPPPHHHPPPPPPPPPPHHPHPHPPHHPPPHPPPHPHPPPHHPPPPPH PPHHPHPPPHPPPPHPPHHPPHPH

9WGA	170	RCGEQGSNMECPNNLCCSQYGYCGMGGDYCGKGCQNGACWTSKRCGS QAGGATCPNNHCCSQYGHCGFGAEYCGAGCQGGPCRADIKCGSQSGGK LCPNNLCCSQWGFCGLGSEFCGGGCQSGACSTDKPCGKDAGGRVCTNN YCCSKWGSCGIGPGYCGAGCQSGGCDA
		
		PHPPPPPPHPHPPPHHHPPHPHHPHPPPHHPPPHPPPPHHPPPPHPPPPPPPP HPPPPHHPPHPPHPHPPPHHPPPHPPPPHPPPHPHPPPPPPPHHPPPHHHPPH PHHPHPPPHHPPPHPPPPHPPPPPHPPPPPPPHHPPPHHHPPHPPHPHPPPHH PPPHPPPPHPP

1RBP	174	ERDCRVSSFRVKENFDKARFSGTWYAMAKKDPEGLFLQDNIVAEFSVDE TGQMSATAKGRVRLLNNWDVCADMVGTFTDTEDPAKFKMKYWGVASF LQKGNDDHWIVDTDYDTYAVQYSCRLLNLDGTCADSYSFVFSRDPNGLP PEAQKIVRQRQEELCLARQYRLIVHNGYC
		
		PPPHPHPPHPHPPPHPPPPHPPPHHPHPPPPPPPHHHPPPHHPPHPHPPPPPHP PPPPPPHPHHPPHPHHPPHHPPHPPPPPPPPHPHPHHPHPPHHPPPPPPPHHH PPPHPPHPHPHPHPHHPHPPPHPPPHPHHHPPPPPPHPPPPPPHHPPPPPPHH HPPPHPHHHPPPHH

### Experiment over data set I

Data set I consists of eight peptides of 20-64 amino acids which have been widely used in previous research [13-18,20,25,26]. In this experiment, we set the crossover rate and mutation rate to be 0.85 and 0.4, respectively. The population size is 10. The iteration size is 30 for sequence 1-5, 100 for sequence 6-7, and 150 for sequence 8. Table 5 compares our results with the results reported by several previous approaches, including ETS [15] which is proposed by Böckenhauer *et al*., [15] to integrate Tabu search, HGA which is a hybrid genetic algorithm proposed by Hoque *et al*. [22], and MA [18,26] which is a memtic algorithm on 2D triangular lattice and extended to 3D FCC in this study. The results show that both our approach and ETS find optimal conformations for all sequences in this data set and achieve the best performance.

**Table 5:** Result for Data Set I and Comparison with ETS, HGA, and MA.

**Seq**.	Native E (HPstruct)	Len	**ETS**[15]	**HGA**[22]	**MA**[26]	Our Method
S1	**23**	20	**23**	29	**23 **(22.53)	**23 **(22.30)
S2	**23**	24	**23**	28	**23 **(22.63)	**23 **(22.10)
S3	**17**	25	**17**	25	**17 **(17.00)	**17 **(17.00)
S4	**38**	36	**38**	50	**38 **(36.70)	**38 **(36.57)
S5	**74**	48	**74**	65	72 (68.50)	**74 **(71.70)
S6	**73**	50	-	59	69 (62.73)	**73 **(66.60)
S7	**130**	60	**130**	114	122 (115.87)	**130 **(124.80)
S8	**132**	64	**132**	98	115 (107.00)	**132 **(126.40)

### Experiment over data set II

Data set II consists of ten peptides of 48 amino acids each. This set of sequences has been a classical benchmark used on the 3D cube lattice model and it was used on 3D FCC lattice recently by Dotu *et al*. [28]. In this experiment the population size is 40 and the iteration size is 150. The crossover rate and mutation rate are 0.85 and 0.4 respectively. The result is given in Table 6. We compare our approach and several approaches proposed by Dotu *et al.*, [28] which combine Tabu search, constraint programming and large neighbor search (LNS). In Table 6, LS denotes Tabu Search with random initialization, LS-G denotes Tabu Search combined with constraint programming, LS-2N denotes2-Neighborhood Tabu Search with random initialization, LS-2N-G denotes2-Neighborhoods Tabu Search combined with constraint programming, LNS-MULT denotes Multiple Sequence Reoptimized LNS, and LNS-3D denotes 3D Structure Reoptimized LNS [28]. The results show that only our approach and LNS-MULT can find optimal conformations for all sequences in this data set.

**Table 6:** Result for Data Set II and Comparison with LNS-based approaches [28].

**Seq**.	Native E (HPstruct)	Len	LS	LS-G	LS-2N	LS-2N-G	LNS-MULT	LNS-3D	Our Method
H1	**69**	48	65 (57.50)	51 (47.17)	68 (64.70)	68 (64.61)	**69 **(66.77)	**69 **(67.68)	**69 **(67.37)
H2	**69**	48	64 (56.59)	55 (46.79)	**69 **(64.32)	68 (62.51)	**69 **(66.60)	**69 **(66.73)	**69 **(66.97)
H3	**72**	48	66 (56.69)	58 (54.38)	68 (62.08)	67 (62.51)	**72 **(68.02)	71 (68.06)	**72 **(68.80)
H4	**71**	48	65 (58.08)	56 (49.26)	67 (63.15)	68 (63.10)	**71 **(67.31)	**71 **(67.61)	**71 **(68.10)
H5	**70**	48	64 (57.01)	57 (42.95)	67 (63.38)	68 (63.79)	**70 **(66.98)	**70 **(67.04)	**70 **(67.77)
H6	**70**	48	63 (56.52)	40 (34.35)	69 (63.38)	68 (64.91)	**70 **(67.49)	**70 **(67.43)	**70 **(66.93)
H7	**70**	48	63 (58.15)	49 (41.10)	68 (63.36)	67 (63.75)	**70 **(66.55)	69 (66.68)	**70 **(67.57)
H8	**69**	48	63 (55.31)	54 (50.27)	67 (62.20)	66 (62.56)	**69 **(65.80)	**69 **(65.81)	**69 **(66.37)
H9	**71**	48	67 (58.91)	54 (46.77)	69 (64.90)	69 (64.40)	**71 **(67.95)	**71 **(67.92)	**71 **(69.10)
H10	**68**	48	64 (57.47)	45 (30.03)	67 (63.96)	67 (63.61)	**68 **(65.76)	68 (65.67)	**68 **(66.47)

### Experiment over data set III and IV

Data set III consists of 15 sequences of length 90-200 which are used in Dotu *et al*. [28]. To our knowledge, no EA-based approaches have been reported for sequences of such length. We compare our approach with the LNS-based [28] and HPstruct [23,24]. It should be pointed out that HPstruct by Will [27] is a sofware tool for the protein structure prediction on the HP lattice model which implements the constraint programming and hydrophobic threading algorithm developed by Backofen and Will [27]. Table 7 summarizes the results. HPstruct finds optimal conformations and out-performs our method, provided that HPstruct converges. However, for sequence F180_1 and F180_2, HPstruct does not return any conformation. As noted in [28] and experienced in our experioment, HPstruct is limited by pre-computed H-cores, and no conformation will be returned if it fails to converge. Our method is able to find conformations for these 2 sequences with energy lower than those obtained by LNS-based approaches [28], including LNS-MULT and LNS-3D which perform better than our approach for the first 12 sequences, but worse for the last 3 sequences.

**Table 7:** Result for Data Set III and Comparison with LNS-based approaches [28].

**Seq**.	Native E HPstruct	Len	LS	LS-G	LS-2N	LS-2N-G	LNS-MULT	LNS-3D	Our Method
F90_1	**168**	90	143 (125.75)	104 (102.97)	154 (142.25)	153 (142.77)	164 (156.83)	**165** (157.39)	161 (151.77)
F90_2	**168**	90	142 (123.68)	117 (112.05)	156 (141.45)	157 (141.89)	**163** (155.05)	163 (155.81)	161 (153.77)
F90_3	**167**	90	138 (121.80)	110 (101.70)	157 (143.79)	159 (145.24)	163 (156.23)	163 (157.20)	**164** (153.13)
F90_4	**168**	90	144 (124.35)	94 (92.74)	162 (144.17)	158 (139.26)	**164** (156.20)	163 (156.54)	159 (152.67)
F90_5	**167**	90	138 (121.59)	110 (107.65)	157 (143.32)	154 (145.00)	163 (155.77)	**164** (157.46)	160 (152.60)

S1	**357**	135	296 (271.03)	276 (270.99)	343 (320.55)	345 (323.81)	349 (332.37)	**351** (336.74)	330 (311.53)
S2	**360**	151	304 (268.43)	250 (244.23)	339 (318.30)	339 (316.60)	349 (328.98)	**353** (334.17)	325 (303.80)
S3	**367**	162	293 (259.55)	234 (228.71)	332 (310.02)	337 (306.03)	351 (323.77)	**353** (329.80)	324 (299.33)
S4	**370**	164	294 (263.73)	226 (222.99)	337 (307.77)	329 (300.92)	346 (323.98)	**354** (334.22)	325 (300.50)

R1	**384**	200	287 (240.85)	212 (205.58)	292 (254.69)	291 (264.53)	313 (287.98)	**330** (305.54)	302 (283.90)
R2	**383**	200	290 (239.12)	209 (205.60)	294 (262.74)	296 (267.75)	331 (289.83)	**333** (308.31)	299 (284.30)
R3	**385**	200	260 (230.57)	228 (212.12)	305 (260.70)	299 (267.05)	325 (288.49)	**334** (307.76)	302 (284.60)

F180_1	**?**	180	244 (204.28)	201 (188.06)	261 (232.30)	265 (240.88)	289 (264.06)	293 (269.07)	**320** (288.41)
F180_2	**?**	180	240 (222.40)	228 (211.07)	279 (255.24)	278 (254.11)	302 (280.84)	312 (287.21)	**321 **(295.50)
F180_3	**378**	180	256 (227.69)	195 (191.91)	292 (262.86)	287 (261.55)	306 (286.78)	313 (295.31)	**316** (294.67)

**Figure 8:**
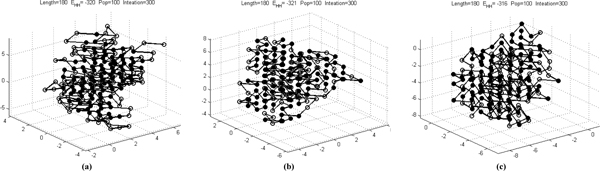
**Configurations for F180_1, F180_2 and F180_3 obtained by our approach**.

In the comparison, our approach performs best for sequence F180_1 and F180_2. Figure 8 gives the conformations returned by our approach for sequences F180_1, F180_2 and F180_3.

**Figure 9:**
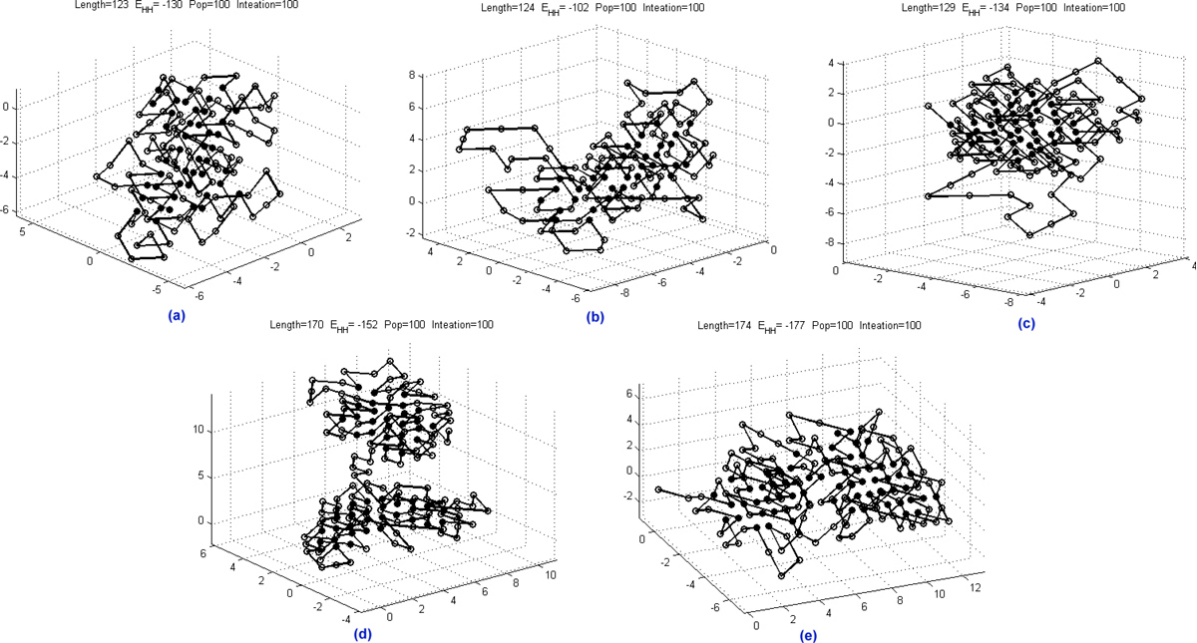
**Configurations for sequences in Data Set IV obtained by our approach**.

We further submit 5 sequences in data set IV, which are selected from PDB with the ID: *4BP2, 2AAS, 5LYZ, 9WGA, and 1RBP*, and used in [45] to study the effect of disulfide bonds in protein structure prediction. HPstruct fails to return any conformation for all 5 sequences. Figure 9 shows the conformations by our approach. The results for data sets III and IV suggest that, although HPstruct performs the best, our approach is more robust than HPstruct and can be used as complemenntary to HPstruct, especially when it fails to converge. In this case, our approach may perform better than the LNS-based approach as shown in the experiments for sequences F180_1 and F180_2.

## Conclusions

In this paper an effective EA-based approach for protein folding is presented; the geometry of the 3D FCC lattice has been investigated and several rotations to enhance crossover have been identified. The well-known Pull Move has been generalized, and a lowering bound method has been developed to reduce the search space of K-site move which is used for mutation. It is shown that the combination of rotation, generalized Pull Move and K-site move can enhance the search performance of traditional EA-based approaches. The approach presented is purely EA-based; it does not rely on any optimization library, can be modified to work with any fitness function, and can be easily integrated with Monte Carlo and Tabu searches. Experiments were carried out over several data sets. Although the results show that HPstruct, which is based on constraint programming, performs better than our approach, provided that HPstruct converges, it failed to converge for several sequences in our experiment. Our approach can be used as complementary to HPstruct, especially when HPstruct fails to converge. In the future, further work can be focussed on experiments to improve the search capability of our algorithm for more data sets, especially for long sequences, as well as for more tedious fitness functions such as 20 amino acid pairwise interaction energy functions. In addition, future work will include the combination of more information, such as disulfide bonds and secondary structures which can be effectively predicted from primary sequences in the search process to find structures which are closer to real native structures.

## Competing interests

The authors declare that they have no competing interests.

## Authors' contributions

JJ proposed the study of lattice rotation and participated in designing the algorithm and writing the manuscript. SC worked out the details, implemented the algorithm, carried out the experiment and drafted the manuscript. Both authors read and approved the final manuscript.

## Declarations

The publication costs for this article were funded by the authors.

This article has been published as part of *Proteome Science *Volume 11 Supplement 1, 2013: Selected articles from the IEEE International Conference on Bioinformatics and Biomedicine 2012: Proteome Science. The full contents of the supplement are available online at http://www.proteomesci.com/supplements/11/S1.
